# Correction: Non-metal cocatalyst CNT-modified emerging g-C_3_N_4_ for enhanced treatment of waste drilling fluid filtrate

**DOI:** 10.1039/d5ra90026c

**Published:** 2025-03-24

**Authors:** Wenzhe Li, Xudong Wang, Ye Chen, Pengcheng Wu, Zhengtao Li, Zhiqiang Wang, Deng Gu

**Affiliations:** a Engineering Technology Research Institute of PetroChina Southwest Oil and Gas Field Company Chengdu 610031 China chenye_2020@petrochina.com.cn dengx202211@163.com; b State Key Laboratory of Oil and Gas Reservoir Geology and Exploitation, School of Oil and Natural Gas Engineering, Southwest Petroleum University Chengdu 610500 China

## Abstract

Correction for ‘Non-metal cocatalyst CNT-modified emerging g-C_3_N_4_ for enhanced treatment of waste drilling fluid filtrate’ by Wenzhe Li *et al.*, *RSC Adv.*, 2025, **15**, 1311–1322, https://doi.org/10.1039/D4RA07393B.

The authors regret that an incorrect version of Fig. 7 was included in the original article. The correct version of Fig. 7 is presented here.

**Fig. 7:**
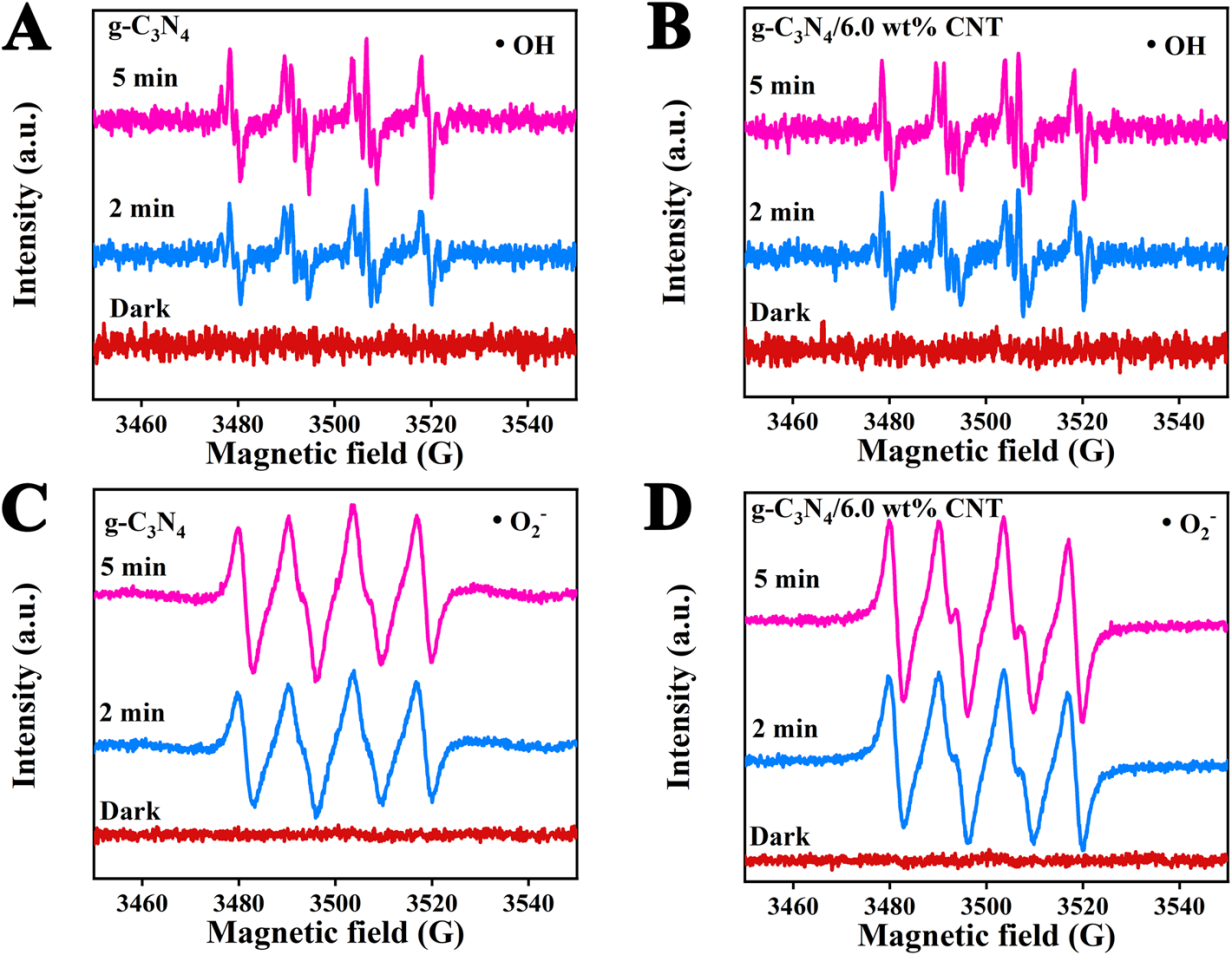
The ESR spectral analysis of active species ˙OH (A and B) and ˙O_2_^−^ (C and D) during photodegradation processes.

The Royal Society of Chemistry apologises for these errors and any consequent inconvenience to authors and readers.

